# Tailoring bilberry powder functionality through preprocessing and drying

**DOI:** 10.1002/fsn3.972

**Published:** 2019-02-19

**Authors:** Lovisa Eliasson, Gabriel Oliveira, Maria Ehrnell, Evelina Höglund, Marie Alminger

**Affiliations:** ^1^ RISE Research Institute of Sweden, Agrifood and Bioscience Gothenburg Sweden; ^2^ Department of Biology and Biological Engineering, Food and Nutrition Science Chalmers University of Technology Gothenburg Sweden

**Keywords:** bilberry, dispersibility, drying, flowability, polyphenols, processing

## Abstract

Berry powders are popular as ingredients in a range of food products, where they naturally provide flavor, color, texture, polyphenols, fiber, and other nutrients. The choices regarding processing techniques and conditions influence the quality attributes of berry powders. The aim of this study was to study the effects on bilberry powder functionalities of applying different preprocessing techniques (purée mixing and juice pressing vs. untreated whole berries) prior to hot air drying and milling. Drying of press cake reduced the drying time by 72% and increased the total apparent phenolic content of the final powder by 44%, as compared to the powder of dried whole berries. The press cake powder showed an easier flowing behavior than the powders from whole berries and puréed berries. Dispersibility (in water and dairy cream) was 60% higher for powders from whole berries and puréed berries, as compared to press cake. The total phenolic content of the dispersed powders was highest for whole berries and puréed berries. Bilberry powder functionality can be modulated through the selection of an appropriate preprocessing technique before drying and milling. This tailors the powder properties into food ingredients ready for different applications, without the need for additives.

## 1 INTRODUCTION

The healthy images associated with berries, in combination with their appreciated sensory attributes, make berries attractive to consumers (Figuerola, 2007; SITRA, 2008). However, the availability of fresh berries is limited to a short season, and they are highly perishable if not further processed. Drying is a commonly used method for extending the shelf‐life of fruit and vegetables, enabling the creation of year‐round, berry‐based food products, and providing added value in terms of health and convenience (Figuerola, 2007; Jangam & Mujumdar, 2010).

Bilberries (*Vaccinium myrtillus *L.) grow wild in the forests and mountain areas of northern Europe and North America, and the berry fruit is popular both as a food delicacy and as a medicinal agent (Morazzoni & Bombardelli, 1996). Bilberries are naturally rich in functional components, such as polyphenols, sugars, organic acids, and dietary fiber (Aura et al., 2015; Mikulic‐Petkovsek, Schmitzer, Slatnar, Stampar, & Veberic, 2015). Laaksonen, Sandell, and Kallio (2010) have identified 58 different phenolic compounds in bilberries, including 15 different anthocyanins. Anthocyanins, which are the principal polyphenols in bilberries, have demonstrated biological activities and potentially contribute to the prevention of a number of diseases, such as diabetes, cardiovascular diseases, and ocular disorders (Pojer, Mattivi, Johnson, & Stockley, 2013). Furthermore, anthocyanins create the typical blue color of bilberries and therefore have value as a natural food colorant (Mukhopadhyay, 2000).

Drying is a time‐ and energy‐consuming process that often requires preprocessing (mechanical, chemical, or thermal) of the raw material for achieving an efficient drying process as well as a satisfactory level of quality of the dried product (Figuerola, 2007). The skin of the berry acts as a barrier to moisture removal, thereby prolonging the drying time. This may result in significant losses of bioactive compounds, entailing negative impacts on the sensory attributes and functional properties of the dried product.

Bilberry press cake, which is a by‐product of juice production, is a suitable starting material for the production of dried berry products, with respect to both drying efficiency and by‐product valorization. Mechanical pressing of the juice increases the dry‐matter content of the berry material, thereby facilitating the drying process (Oszmiański, Wojdyło, & Lachowicz, 2016). However, together with removal of juice, water‐soluble components, such as sugars, acids, and bioactive compounds, are lost, which alters the natural composition of the berry material (Laaksonen et al., 2010).

Berry ingredients, such as powders, are important drivers of the berry market due to their convenient format for both consumers and the food industry (SITRA, 2008). The powder form enables a range of applications in, for example, extruded products, bakeries, sauces, beverages, ice creams, yogurts, and confectionary (Karam, Petit, Zimmer, Djantou, & Scher, 2016). Berry powders can be prepared from whole berries, puréed berries, juice, berry by‐products, and extracts (Chakraborty, Savarese, Harbertson, Harbertson, & Ringer, 2009; Oszmiański et al., 2016). Depending on the raw material composition and choice of processing technique, opportunities arise to tailor the berry powder functionality. Approaches to contribute to a more sustainable agro‐food production chain, for example, by use of technologies with less use of energy and with better preservation of nutrients and functional properties are urgently needed. Despite the large number of studies carried out on the utilization of the huge amounts of co‐products generated from berry processing, relatively few processes have yet found industrial application. Thus, there are environmental, nutritional, and economic reasons to develop new knowledge and enhance opportunities for upgrading of processing solutions to preserve the quality attributes of berry powders.

The objective of this study was to investigate the effects on bilberry powder functionalities of introducing different preprocessing modalities prior to hot air drying and milling. The effects on the overall quality of the bilberry powder of mixing berries into a purée and preparing a press cake by mechanically pressing away the juice, before drying and milling, were compared with bilberry powder prepared from whole berries. The drying time and the different characteristics in terms of water activity, moisture content, particle size distribution, flowability, and total phenolic content of the bilberry powders were determined. The potential of bilberry powders as ingredients in liquid food products was studied by evaluating the dispersibility of the powders in both water and cream. This is the first part of a two‐step study where the bilberry powder functionality is tailored by effects from the whole production process including pretreatments, drying techniques, and post‐treatments. In the second part, Oliveira et al. (2019) describe the effects of drying and fractionation on the stability of polyphenols and anthocyanins in bilberry press cake powder.

## 2 MATERIALS AND METHODS

Bilberries (*Vaccinium myrtillus *L.) with moisture content of 873 ± 5.6 g/kg were supplied by Olle Svensson AB (Olofström, Sweden) and stored in the dark at −40°C prior to the experiments.

### 2.1 Preprocessing of bilberries

Hot air drying was applied to the whole berries, puréed berries, and press cake. Whole berries were thawed in the dark at room temperature for 2 hr, before drying. The purée was prepared by mixing 280 g of whole berries in duplicate in a mini chopper (C3; Empire Sweden AB, Bromma, Sweden) for 40 s. Before drying, the purée was distributed into medallions (diameter of 4 cm, each containing 9 g purée) on a silicon carpet. The press cake was prepared by mechanical pressing (Hafico, Germany) of 900 g of berries that were thawed in the dark at 6°C for 16 hr. The pressing was performed three times, and the resulting press cake was combined into a single sample. A maximum pressure of 200 kg/cm^2^ was applied, giving a juice yield of 813 ± 6.1 g/kg.

### 2.2 Hot air drying

Drying was carried out in a hot air oven (Garomat 142; Electrolux AB, Stockholm, Sweden) with an air velocity of 6.1 m/s. Bilberries (whole, puréed, or press cake) were distributed into two trays (21 cm × 30 cm), each containing 250 g of material, and dried at 40°C protected from light until a water activity of about 0.5 was achieved. The thickness of the materials before drying was 0.85 ± 0.18 cm for whole berries, 0.69 ± 0.07 cm for puréed berries, and 0.99 ± 0.29 cm for press cake. The drying times required for each sample (whole berries, puréed berries, and press cake) were determined by assessing on a continuous basis the decrease in material weight and the water activity during pretrials. The dried material was stored in sealed polyamide/polyethylene plastic pouches and protected from light at −40°C until milling.

### 2.3 Milling

Each berry material (dried whole berries, puréed berries, and press cake) was milled in triplicate (75 g × 3) in a knife mill (Grindomix GM 200; Retsch GmbH, Haan, Germany) at 7,500 rpm for 30 s. After milling, the bilberry powders from the triplicates were combined into a single sample and stored in sealed polyamide/polyethylene plastic pouches at −80°C until analysis.

### 2.4 Moisture content and water activity

The water activity was measured in triplicate using the Aqua Lab 4TE (Decagon Devices, Pullman, WA, USA). The moisture content was determined gravimetrically in a vacuum oven (Sanyo Gallenkamp, Loughborough, UK) by weighing about 2 g of powder into an aluminum dish for drying at 70°C and 90 kPa, until a constant weight was reached.

### 2.5 Particle size distribution

Determination of the particle size distribution of the powders was achieved using a vibratory sieve shaker (Analysette 3; FRITSCH GmbH, Idar‐Oberstein, Germany) with sieves of mesh sizes 250 µm, 500 µm, and 710 µm. Sieve shaking was performed for 10 min at an amplitude of 1.5 mm and an interval time of 10 s. Duplicate measurements were made with 20 g powder used each time.

### 2.6 Flowability

A powder flow tester (Brookfield Engineering Laboratories Inc., Middleboro, MA, USA) was used to determine the flowability of the bilberry powders. The flowability was expressed as the powder flow function, which is the best‐known indicator of powder flowability. The powder flow function, which represents a plot of the unconfined failure strength versus the major principal consolidation stress, gives a measure of the strength that the powder retains at a stress‐free surface at a given stress level. The measurements were performed over five major principal consolidation stresses of approximately 1.6, 3.3, 6.9, 14.0, and 28.2 kPa. The bilberry powder was loaded into a volume shear cell of 38 cm^3^, and a vane lid was used to compress the sample during the analysis. Measurements were run in triplicate using a standard flow function test program (Powder Flow Pro V1.2 software). The flowability of the powder was classified according to the flow factor index (ff): nonflowing, ff < 1; very cohesive, 1 < ff < 2; cohesive, 2 < ff < 4; easy‐flowing, 4 < ff < 10; and free‐flowing, 10 < ff (Schulze, 2008).

### 2.7 Dispersibility

In the present study, dispersibility is defined as the amount of particles, and the proportional number thereof, that remains suspended in the liquid phase after mixing of the powder with a liquid and subsequent resting time. The weights of the dissolved compounds are also considered in the liquid phase. The dispersibility levels of the bilberry powders were studied in water and dairy cream (150 g/kg fat), using a method adapted from those used previously (Jaya & Das, 2004; Shittu & Lawal, 2007). Briefly, 5 g of dry powder was added to 100 g liquid (tap water or dairy cream) and stirred for 5 min using a magnetic stirrer (RCT basic; IKA‐Werke GmbH, Staufen, Germany) and a magnet that covered the bottom of a 400 ml of glass beaker. After stirring, the suspension was left to rest for 12.5 min, to allow the suspended particles to settle, and thereafter, 7 g of the supernatant was carefully pipetted into a petri dish. The petri dishes with supernatants were placed in a vacuum oven (Fistreem International Ltd., Loughborough, UK) at 80°C and 90 kPa, and dried until a constant weight was reached. Samples of the cream without added powder were dried in the same way, so as to subtract the weight of the cream components, thereby allowing determination of the percentage of dispersed particles (w/w). The cream was obtained from a local supermarket, and the drying was carried out in duplicate for each sample. A separate dispersibility experiment was performed to acquire samples for determination of the total phenolic content of the supernatant. To obtain sufficient material for this analysis, 10 g of dry powder was added to 200 g liquid (water or cream).

The pH (SevenGo Duo pro SG78; Mettler Toledo, Columbus, OH, USA) and %Brix (HI96801; Hanna Instruments, Woonsocket, RI, USA) of the supernatant were measured for powders that were dispersed in water.

### 2.8 Total phenolic content

The total content of phenolic compounds was determined according to the methods described previously (Barnes, Nguyen, Shen, & Schug, 2009; Howard, Clark, & Brownmiller, 2003). Freeze‐dried bilberry powder and samples from the dispersibility experiments (0.2 ± 0.015 g) were extracted in triplicate with acidified methanol [MeOH:H_2_O (70:30 mix) plus 1% trifluoroacetic acid]. After the addition of Folin–Ciocalteu reagent, the extracts were analyzed spectrophotometrically against a standard curve of gallic acid. The total phenolic content (TPC) is presented as gallic acid equivalents (GAE) per kg of dry weight (g GAE/kg DW).

### 2.9 Statistical analysis

One‐way analysis of variance (ANOVA) and *Tukey's* honestly significant difference (HSD) test were applied to compare the samples. Statistical significance was defined as *p* < 0.05. Statistical analysis was performed using the IBM^®^ SPSS^®^ Statistics ver. 24 software package.

## 3 RESULTS AND DISCUSSION

### 3.1 Effects of preprocessing on drying time and moisture content

As expected, the choice of preprocessing technique strongly influenced the drying time required to attain a water activity of about 0.5. Whole untreated berries showed the longest drying time of 27 hr, followed by 15 hr for the puréed berries and 7.5 hr for the press cake (Table 1). The waxy skin of whole berries acts as a barrier during drying, retarding moisture removal. By blending the berries to a purée, the drying time was reduced by 44%, due to extensive disruption of the bilberry skin. The press cake had a lower moisture content from the start (693 g/kg), as compared to the whole berries (873 g/kg) and puréed berries (873 g/kg), which explains the more rapid drying of the press cake. In addition, mechanical pressing ruptures the bilberry skin, thereby facilitating moisture removal. Together with the juice, sugars and acids likely disappear from the press cake as described by Laaksonen et al. (2010). A lower sugar content of the press cake, reducing its hygroscopicity, could also facilitate moisture removal during drying (Oszmiański et al., 2016).

**Table 1:** Moisture content (g/kg), drying time (h), and water activity of powder from dried whole berries, puréed berries, and press cake

Sample	Drying time (h)	Moisture content (g/kg)	Water activity
Whole berry powder	27	132 ± 1.1	0.49 ± 0.001
Puréed berry powder	15	142 ± 0.7	0.50 ± 0.001
Press cake powder	7.5	78 ± 0.2	0.50 ± 0.003

The moisture contents of the whole berry and puréed berry powders were 40.9%–45.1% higher than those of the press cake powder, although the water activity for all the powders lays within the range of 0.49–0.50 (Table 1). The higher moisture contents of the whole berry and puréed berry powders could possibly be connected to higher sugar contents in these samples compared to the press cake powder in which these components likely have been removed together with the juice (Laaksonen et al., 2010). Sugars bind water, thereby reducing the proportion of free water and lowering the water activity (Sandulachi, 2012). Pretreatment of raspberries in different sugar and acid solutions before drying has previously been shown to influence the equilibration between moisture content and water activity in dried raspberries (Settea, Salvatoria, & Schebor, 2016).

### 3.2 Effects of preprocessing on particle size distribution

**Figure 1:**
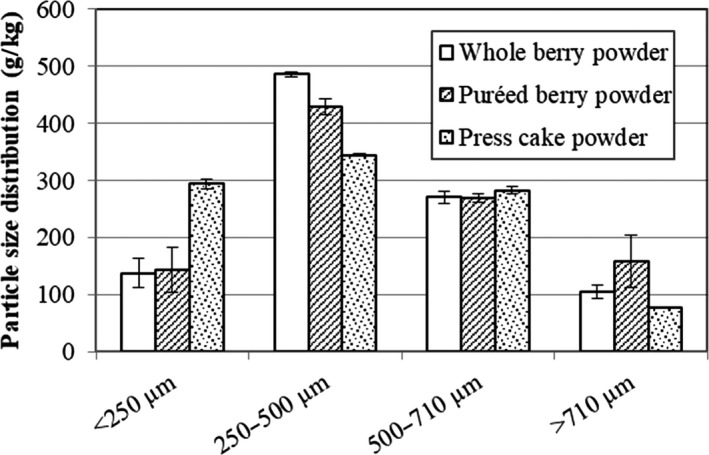
Particle size distribution (g/kg) of bilberry powders from dried whole berries, puréed berries, and press cake

The particle size distributions of food powders are of industrial importance because of their influences on various properties, such as flowability, bulk density, and compressibility (Barbosa‐Cánovas, Harte, & Yan, 2009). A similar pattern of particle size distribution was noted for all the bilberry powders, with some differences. As shown in Figure 1, press cake powder had the highest proportion of the smallest size (<250 μm) fraction with 294 g/kg compared to 138 g/kg and 143 g/kg for whole berry and puréed berry powder, respectively. In contrast, the whole berry (486 g/kg) and puréed berry powders (429 g/kg) showed a higher proportion of the size fraction 250–500 μm compared to press cake powder (345 g/kg). After drying, the press cake was lower in moisture content than the whole berries and puréed berries, which likely rendered the material more friable and therefore easier to mill into smaller particles. Also, differences in sugar content, and shape and size of the dried materials before milling could have influenced the final particle size.

### 3.3 Effects of preprocessing on flowability

**Figure 2:**
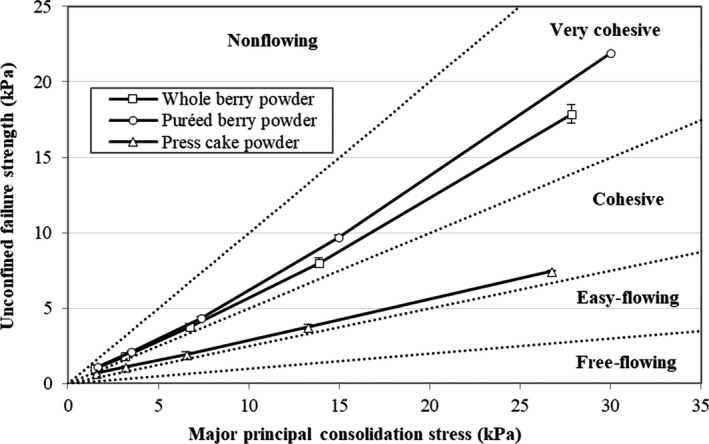
Flow function of bilberry powders from dried whole berries, puréed berries, and press cake, at different levels of major principal consolidation stress

The flow behaviors of food powders are crucial parameters for handling and processing operations, such as storage, transportation, mixing, and packaging, and they are affected by, for example, moisture content, composition, particle size, and particle structure (Teunou, Fitzpatrick, & Synnott, 1998). The flow functions (Figure 2) of both the whole berry and puréed berry powders showed a highly cohesive behavior, while the press cake powder was cohesive on the cross section to easy‐flowing. A powder becomes more cohesive with increasing moisture content due to the formation of liquid bridges between the particles (Teunou et al., 1998). Therefore, the lower moisture content of the press cake powder (Table 1) makes it easier flowing than the other powders. Furthermore, sugars have likely been removed from the press cake with the juice as described by Laaksonen et al. (2010). A reduced sugar content in the press cake powder could have reduced its cohesiveness. The presence of sugars and acids increases the stickiness of the powder by reducing its glass‐transition temperature, thereby resulting in a less‐free‐flowing powder (Jaya & Das, 2004; Kim & Kerr, 2013).

The puréed berry powder showed a more cohesive behavior than the whole berry powder, which may reflect its slightly higher moisture content (142 ± 0.7 g/kg vs. 132 ± 1.1 g/kg). That the puréed berry undergoes a higher degree of tissue disruption is another possible explanation. Excessive tissue disruption and leaching of sugars to the surface increase the risk of glass‐transition problems and stickiness when drying sugar‐rich fruits (Bhandari & Howes, 1999).

From the industrial point of view, the highly cohesive flow behaviors of the whole berry and puréed berry powders could cause problems during handling and processing operations, and the addition of a high‐molecular‐weight drying aid might be needed. Kim and Kerr (2013) produced dried powders from whole blueberry fruit and found that it was necessary to add maltodextrin to reduce stickiness and thereby improve flowability. To avoid the use of additives such as maltodextrin, press cake is preferred as the starting material for bilberry powder production, as it naturally results in a more‐easy‐flowing powder.

The particle size of a powder strongly influences its flowability. In a finely milled powder, there is an increased risk of cohesion and the flowability is reduced. Teunou et al. (1998) have reported that powders with particles sizes larger than 200 μm are, in general, free‐flowing. Therefore, the flowability characteristics of the bilberry powders generated in this study can probably be improved by applying a less‐extensive milling regimen, resulting in larger particle sizes.

### 3.4 Effects of preprocessing on dispersibility

Bilberry powders are suitable as ingredients in liquid food products, such as beverages, milk drinks, yogurt, and ice cream, as they provide texture, color, nutrients, and flavor. Therefore, the dispersibility of the bilberry powders was evaluated in two different liquids, water and cream. As bilberry powders are high in insoluble dietary fiber, dispersibility was considered to be a more relevant quality index than solubility for these types of powders (Aura et al., 2015). Dispersibility is a measure on how readily a powder is distributed as a single particle in a liquid phase, in comparison with its solubility when complete dissolution occurs (Shittu & Lawal, 2007). Powders with low dispersibility frequently cause problems related to high sedimentation volumes in the liquid, and this negatively influences the food quality (Park, Imm, & Ku, 2001).

**Figure 3:**
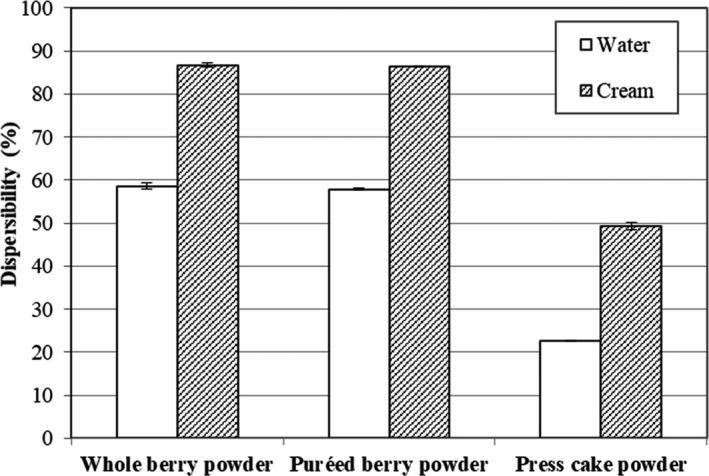
Dispersibility (%, w/w) of bilberry powders from dried whole berries, puréed berries, and press cake in water and cream (150 g/kg fat)

The whole berry and puréed berry powders showed a similar degree of dispersibility, whereas the press cake powder showed 43%–61% lower dispersibility dependent on the type of liquid used (Figure 3). This result is likely connected to the removal of water‐soluble components, such as low‐molecular‐weight sugars and organic acids, together with the juice in the pressing step (Laaksonen et al., 2010). That the press cake powder had lower contents of sugar and acids was indicated by measuring the pH and %Brix values of the supernatants of the powders dispersed in water. The pH values of the berry powders dispersed in water were 3.0 for the whole berry and puréed berry powders, and 3.4 for the press cake. The %Brix values of the supernatants from the whole berry and puréed berry powders were about 10‐fold higher (3.3 vs. 0.3) than those obtained for the press cake. Sugars and acids may leak out from the powder particles and solubilize in the liquid. Particles that contain sugars are probably also more easily dispersed in the liquid due to the water‐binding properties of sugars, thereby increasing the dispersibility of the whole berry and puréed berry powders, whereas particles with a low content of sugars readily sediment to the bottom of the liquid. Shittu and Lawal (2007) have shown that sugar is one of the most important factors influencing the reconstitution properties, such as dispersibility, of cocoa powder.

For all the types of bilberry powders, dispersibility was higher in cream than in water. Cream is an emulsion of fat globules in water, and it contains proteins that are in a colloidal suspension in the aqueous phase (Coultate, 2009). It is likely that cream constituents (fat globules, proteins, and carbohydrates) entrap the bilberry particles, preventing their sedimentation, which explains the increased dispersibility of the particles in cream.

### 3.5 Effects of preprocessing on total phenolic content

The press cake powder showed the highest TPC (Table 2), which is explained by the higher TPC of the starting material before drying, owing to a concentration effect when the sugars and acids were removed with the juice in the pressing step (Laaksonen et al., 2010). This is in agreement with Oszmiański et al. (2016), who found that the use of pomace from honeysuckle berries increased the total polyphenol content of berry powders, as compared to the use of whole berries. In addition, mechanical disruption of cell walls during pressing may have improved the extraction efficiency during the TPC analysis.

**Table 2:** Total phenolic content (g/kg) in bilberry powder from dried whole berries, puréed berries, and press cake, compared to whole berries and press cake before drying

Sample	Total phenolic content (g GAE/kg DW)
Whole berries before drying	21.38^b^ ± 3.81
Press cake before drying	36.48^a^ ± 2.96
Whole berry powder	21.9^b^ ± 1.15
Puréed berry powder	26.3^b^ ± 2.53
Press cake powder	39.2^a^ ± 1.10

Notes

Values followed by the same letter were not significantly different (*p* < 0.05) based on Tukey's test.

DW: dry weight; GAE: Gallic acid equivalent.

No reduction in TPC was observed during drying for either the press cake or the whole berries, although the drying time was up to 27 hr for the whole berries. Skoczeń‐Słupska, Gębczyński, and Kur (2016) reported a TPC reduction of 23% when drying at 60°C for 10 hr bilberries that had an initial TPC of 50 g/kg DW. Michalczyk, Macura, and Matuszak (2009) used a bilberry batch with an initial TPC of about 70 g/kg DW and observed a reduction in TPC of about 22% after drying at 40°C for 72 hr. However, comparison of the impacts of various processing methods on the stability levels of bioactive compounds is difficult due to the use of different processing conditions, including pretreatment and storage conditions, as well as the use of different bilberry varieties. Nevertheless, the air‐drying temperature is one of the most important factors determining the quality (e.g., content of bioactive compounds and sensory attributes) of the end‐product (Chen & Martyrnenko, 2013; Larrauri, Rupérez, & Sauro‐Calixto, 1997). The lower initial TPC of the fresh material (21 g/kg DW) in the present study, as well as differences in drying time and temperature, may explain why the reduction in TPC reported in previous studies has been more pronounced. Skrede, Wrolstad, and Durst (2000) found that the stability of anthocyanins in blueberries was influenced to different extents during processing dependent on the type of anthocyanin structure. The presence of more stable polyphenols in the present study may therefore also have contributed to the TPC preservation.

**Figure 4:**
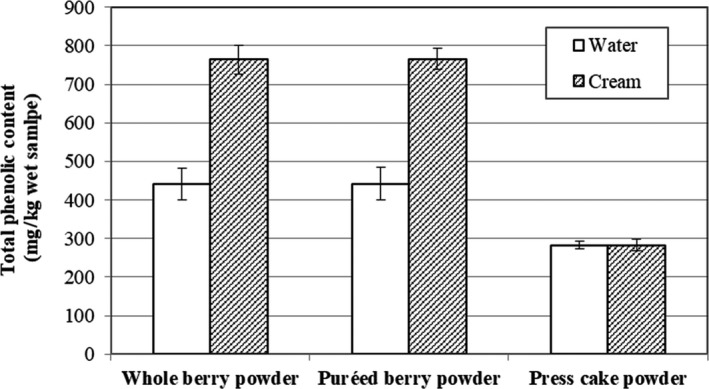
Total phenolic content of dispersed bilberry powders in water and cream expressed as mg GAE/kg wet sample

The TPC values of the dispersed powders (Figure 4) follow a pattern similar to those of the dispersibility results shown in Figure 3, with the exception of the press cake powder, for which the TPC is similar in both water and cream, although the powder dispersibility is twofold higher in cream than in water. This may be linked to a different composition of the press cake powder compared to whole berry and puréed berry powders, with press cake powder having a higher TPC (Table 2) and likely lower levels of sugars and acids as indicated by the %Brix and pH measurements. A large proportion of the polyphenols from the press cake powder probably dissolve in water, while powder constituents, such as seeds, contain fewer polyphenols and sediment out. In cream, the seeds are supported by the matrix, so the difference in the TPC of the dispersed powder is reduced between water and cream. The presence of sugars and acids, which easily dissolve in water, in the whole berry and puréed berry powders probably diminishes the effect for these powders. Whole berry and puréed berry powders dispersed in cream gave the highest TPC per kg wet sample (Figure 4), which can be related to the high dispersibility values (86%–87%) of these powders (Figure 3).

Due to the presence of other constituents (fat, protein, and carbohydrates) in cream, the dispersed TPC expressed per DW (Table 3) is lower in cream (0.95–2.81 g/kg DW) than in water (14.64–24.14 g/kg DW), where only dry matter from the bilberry powder is present. The dispersion of bilberry powders negatively influences the content of phenolic compounds (Table 3), as compared to the initial powder (Table 2). After dispersion in water, the reduction was in the range of 33%–40% compared to the initial powder. It is possible that there is degradation of phenolic compounds due to exposure to light and moisture. In addition, the TPC may be different for particles that sediment to the bottom and particles that are dispersed in solution.

**Table 3:** Total phenolic content of dispersed bilberry powders in water and cream expressed as g/kg dry weight

Sample	Total phenolic content (g GAE/kg DW)	
	Water	Cream
Whole berry powder	14.64^b^ ± 1.35	2.81^c^ ± 0.14
Puréed berry powder	15.93^b^ ± 1.57	2.81^c^ ± 0.10
Press cake powder	24.14^a^ ± 0.89	0.95^d^ ± 0.05

Notes

Values followed by the same letter were not significantly different (*p* < 0.05) based on Tukey's test.

DW: dry weight; GAE: Gallic acid equivalent.

## 4 CONCLUSIONS

The present study shows that the functionality of bilberry powders can be tailored in a gentle way by applying different mechanical preprocessing techniques. This is done without the use of any additives, organic solvents, or high temperatures. The functionalities of bilberry powders are clearly influenced by the choice of preprocessing technique before drying and milling. Pressing out the juice, creating a press cake, prior to drying reduces considerably the drying time and boosts the TPC of the powder, as compared to whole berry and puréed berry powders. Furthermore, using press cake as the starting material for drying is beneficial in terms of producing a powder with improved flowability. In contrast, powders produced from whole berries and puréed berries show improved dispersibility and higher TPC for the model food product (powders dispersed in water and cream). In summary, by selecting appropriate preprocessing techniques it is possible to adapt the functionalities of bilberry powders, thereby ensuring the desired properties for various end‐uses, without the use of additives.

## ETHICAL STATEMENT

This study does not involve any human or animal testing.

## CONFLICT OF INTEREST

None declared.
